# Liquid–Vapor Coexistence and Spontaneous Evaporation at Atmospheric Pressure of Common Rigid Three-Point Water Models in Molecular Simulations

**DOI:** 10.1021/acs.jpcb.3c08183

**Published:** 2024-03-01

**Authors:** Patrick K. Quoika, Martin Zacharias

**Affiliations:** Center for Functional Protein Assemblies, Technical University of Munich, Ernst-Otto-Fischer-Str. 8, Garching 85748, Germany

## Abstract

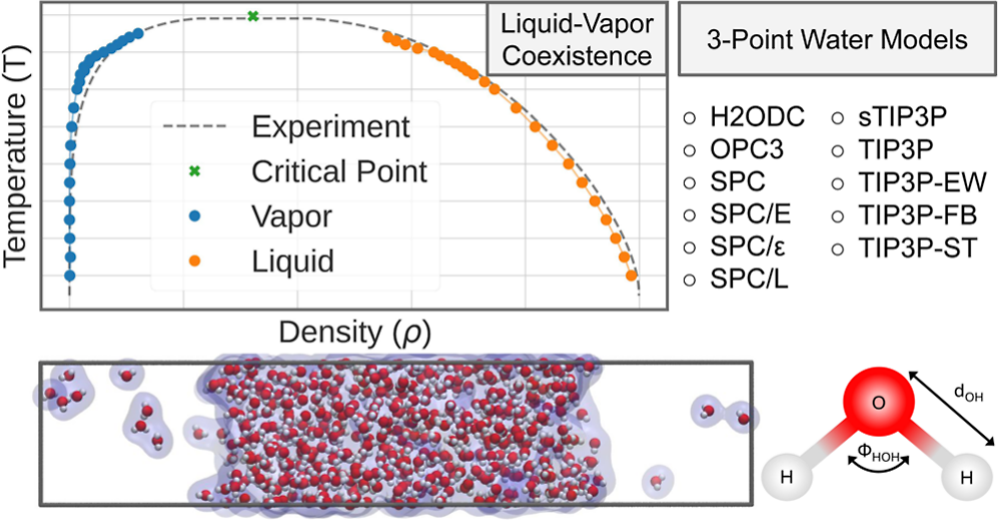

Molecular dynamics (MD) simulations are widely used to investigate molecular systems at atomic resolution including biomolecular structures, drug–receptor interactions, and novel materials. Frequently, MD simulations are performed in an aqueous solution with explicit models of water molecules. Commonly, such models are parameterized to reproduce the liquid phase of water under ambient conditions. However, often, simulations at significantly higher temperatures are also of interest. Hence, it is important to investigate the equilibrium of the liquid and vapor phases of molecular models of water at elevated temperatures. Here, we evaluate the behavior of 11 common rigid three-point water models over a wide range of temperatures. From liquid–vapor coexistence simulations, we estimated the critical points and studied the spontaneous evaporation of these water models. Moreover, we investigated the influence of the system size, choice of the pressure-coupling algorithm, and rate of heating on the process and compared them with the experimental data. We found that modern rigid three-point water models reproduce the critical point surprisingly well. Furthermore, we discovered that the critical temperature correlates with the quadrupole moment of the respective water model. This indicates that the spatial arrangement of the partial charges is important for reproducing the liquid–vapor phase transition. Our findings may guide the selection of water models for simulations conducted at high temperatures.

## Introduction

Molecular dynamics (MD) simulations allow the study of molecular motions at atomistic spatial resolution and picosecond time resolution.^1^ The method is used in materials science, molecular and structural biology, and drug development, among others.^2^ In most cases, MD simulations are performed in aqueous solution with explicit molecular models of the solvent.^3−5^

The design of explicit computational models of water is nontrivial, because of its complex properties.^6^ Thus, in the past decades, a variety of design approaches and multiple generations of water models have been published. Developers of water models are usually confronted with the problem of finding a balance between accuracy and computational cost. Models with different numbers of interaction sites exist: three-point,^7,8^ four-point,^9−14^ five-point,^15,16^ six-point,^17^ even seven-point water models have been developed.^18^ Furthermore, apart from rigid models—i.e., models with fixed bond angle and bond distances—flexible models have also been proposed.^19^ In addition, as an alternative to fixed point-charge models, polarizable water models have been developed.^20−23^ While these models, oftentimes exhibit superior accuracy, they lead to significantly increased computational effort.^24^ Yet another approach to model water in molecular simulations are ab initio methods.^25−27^ Such methods—e.g., Car–Parrinello MD or the Born–Oppenheimer MD approach^28,29^—incorporate polarization (and charge transfer) effects by reevaluating the electron density around the nuclei during every time step in the simulation, by means of quantum mechanical (QM) calculations, such as density functional theory.^30^ Consequently, these methods are potentially very accurate, but require exceedingly high computational effort.^26^ Besides that, the results may be biased due to the choice of QM methodology and other simulation parameters.^31−33^ The most efficient and, thus, still most widely used atomistic water models are rigid three-point water models.^34^

Most developments of molecular models of water aim to reproduce the properties of water at room or body temperature and atmospheric pressure.^12^ However, frequently, realistic simulations at higher temperatures are also desired. This concerns, for example, temperature-dependent processes such as protein denaturation or conformational transitions of thermoresponsive polymers.^35−39^ The choice of water model may influence such temperature-dependent processes in molecular simulations tremendously.^40−42^ In simulations at elevated temperatures, it is important to be sure that the water model of choice is still in the liquid phase. Furthermore, exploring the correlation between the temperature-dependent properties of in silico water and the parameters of different existing models (such as geometry and charge distribution) is of interest.

Here, we investigate the evaporation behavior of various rigid three-point water models in molecular simulations and their liquid–vapor coexistence. First, we estimate the critical temperature and critical density. Furthermore, we estimate the temperature of spontaneous evaporation and the point of maximum density of these models at ambient pressure (1 bar). We only investigate rigid three-point water models since models of this type are still most popular due to their computational efficiency. Lastly, we look for correlations between the water model parameters and the resulting points in the phase diagram.

## Materials and Methods

### Investigated Water Models

**Figure 1:**
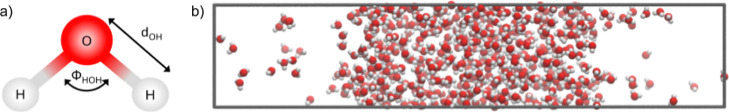
Model geometry of three-point water models (a). Snapshot from a liquid–vapor coexistence simulation (b). This snapshot has been extracted from a simulation with the SPC/ϵ water model at *T* = 550 K. Depending on the simulation temperature, the densities in the liquid and vapor phases vary.

We investigated 11 common rigid three-point water models. These water models are all constructed using the same overall scheme, i.e., TIP3P-type, and may therefore be directly compared in terms of geometrical parameters, partial charges, and Lennard-Jones (LJ) interaction parameters. The studied models are listed in Table 1, including the respective model parameters, for comparison. The general geometry of such water models is visualized in Figure 1. In addition, we provide properties of these water models that may directly be calculated from the model parameters (specifically, the dipole moment and the quadrupole moment) in Table S1. We recognize in the list of analyzed water models that roughly half of the models are older than 2010, whereas the others are more recent. Below, we refer to the latter group as the *modern water models*.

**Table 1:** Model Parameters of the Here-Used Water Models, Given in Alphabetical Order^a^

	*d*_OH_	ϕ_HOH_	σ_O_	ϵ_O_	*q*_H_	year	ref
H2ODC	0.9580	109.47	3.184	0.14173	0.45495	2012	(43)
OPC3	0.9789	109.47	3.17427	0.16341	0.4476	2016	(44)
SPC	1.0	109.47	3.16557	0.1554	0.41	1981	(8)
SPC/E	1.0	109.47	3.16557	0.1554	0.4238	1987	(45)
SPC/ϵ	1.0	109.45	3.1785	0.1687	0.4245	2015	(46)
SPC/L	1.1	104.5	3.1487	0.16049	0.34425	2002	(47)
sTIP3P*	0.9572	104.52	3.1507	0.1521	0.417	1998	(48)
TIP3P	0.9572	104.52	3.1507	0.1521	0.417	1981	(7)
TIP3P-EW	0.9572	104.52	3.188	0.102	0.415	2004	(49)
TIP3P-FB	1.0118	108.15	3.178	0.15583	0.424	2014	(13)
TIP3P-ST	1.023	108.11	3.19257	0.14386	0.42556	2019	(14)

a Bond length *d*_OH_ and LJ parameter σ_O_ are given in [Å]. The LJ parameter ϵ_O_ is given in [kcal/mol] and the bond angle ϕ_HOH_ is given in [°]. (*sTIP3P is the adaptation of TIP3P for the CHARMM force field. The only difference is that sTIP3P has an additional weak repulsive interaction site on the hydrogen atoms.)

Almost all water models have, in parts, been parameterized on some properties of water under (close to) ambient conditions, i.e., around *p* = 1 bar and *T* = 300 K. Which particular properties were in the focus of the parameterization process depends on the particular model. The reader is referred to the original publication for details on the parameterization of each model. Nevertheless, we provide short summaries for all these models in the Supporting Information, Section A. A general exception in terms of the parameterization procedure is OPC3: the authors focused first and foremost on the charge distribution of the model. The LJ parameters were adapted to yield agreement in the radial distribution function with experiment. They did not include any experimental properties in the parameterization procedure but only validated their model against experimental values.

#### Model Parameters

Here, we tested 11 water models, which are listed in Table 1, ordered alphabetically, with the original references. Aspects of the parameterization procedure of these models are given in the Supporting Information, Section A, including a breakdown of the respective acronyms. For convenience, we also visualized the ranking of these properties of all water models in Supporting Information Section B.

#### Derived Properties

Based on the spatial arrangement of the partial charges, we calculated the dipole moment, μ, according to eq 1.^50^ Furthermore, we calculated the quadrupole moment of the water models. Generally, the quadrupole moment is a tensor. However, for water, it may be approximated by a single scalar number, i.e., the tetrahedral quadrupole moment, *Q*_T_.^16,51^ Throughout this article, we refer to *Q*_T_ simply as *the quadrupole moment*. For three-point water models, *Q*_T_ may conveniently be calculated according to eq 2.^12,52^
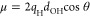
1
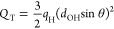
2The above equations are expressed in terms of the angle θ = ϕ_HOH_/2. Both quantities—μ and *Q*_T_—are known to be important to reproduce the experimental behavior of water accurately.^50^ Also, *Q*_T_ is important to obtain the tetrahedral structure of water.^53^

### Simulation Protocol

Two scenarios were investigated. First, we performed liquid–vapor coexistence simulations in the canonical ensemble at various temperatures. From these simulations, the coexistence densities and, ultimately, the critical point of the respective water model can be obtained. Furthermore, we performed canonical simulations of pure liquid and vapor to estimate the enthalpy of evaporation. Second, we performed equilibrium simulations in the isobaric–isothermal ensemble at various temperatures. From these simulations, we obtained equilibrium densities of the respective water models at various temperatures at a pressure of 1 bar. Above a model-dependent temperature—which we call *T*_evap_—we observed spontaneous evaporation in these simulations. Both approaches are described in detail below. For clarity, the temperature of spontaneous evaporation is not equal to the boiling temperature at a given pressure. This is further discussed in the Results and Discussion section.

Generally, we performed the simulations with a rather common setup, using the software GROMACS.^54^ A time step of 2 fs was used in all of the simulations. We treated long-range electrostatic interactions with the particle mesh Ewald method^55^ and long-range LJ interactions with a single cutoff. The cutoff distance was set to 8.5 Å (optimized by GROMACS) for simulations in the liquid phase. We increased the cutoff for vapor-phase simulations to improve the computational efficiency (see below). The velocity-rescale algorithm was used for temperature coupling in all simulations.^56^

#### Liquid–Vapor Coexistence Simulations

To obtain the densities of water vapor and liquid water at various temperatures, we performed simulations with coexisting phases. To this end, we followed a common approach, as described by Muniz et al.^57^ In summary, an initial configuration with a rectangular simulation box, which was extended in the *z* direction, measuring 20 × 20 × 100 Å was prepared. This box contained 512 water molecules, of which 256 were arranged in a density that corresponds to the liquid phase, and the remaining 256 were arranged in a much lower density, corresponding to the vapor phase. A representative snapshot of such coexistence simulation is visualized in Figure 1. We equilibrated these boxes in the canonical ensemble at various temperatures for all of the studied water models. To improve the accuracy of our predictions, we decided to simulate significantly longer than in previous studies by Muniz et al.^57^ and equilibrated the systems for 5 ns followed by a production run of 20 ns. For the subsequent analysis, we separately analyzed these 20 ns in splits of 5 ns. Thereby, we estimated the uncertainty of our estimations.

From the coexistence curves, the densities of liquid and vapor in the respective thermodynamic state can be obtained. To this end, we centered the liquid phase in the simulation box and quantified the mean density of water along the *z*-axis. For the centering procedure, the liquid phase needs to be wrapped eventually (if the liquid slab diffuses across the periodic box). This centering (and the correct wrapping of the periodic images) is crucial to obtain clean data and becomes increasingly difficult for temperatures close to the critical temperature for two reasons: first, the liquid and vapor densities get closer; and second, the liquid phase diffuses quicker and thus may cross the periodic borders more frequently, especially for long simulation times. We fitted these densities with a sigmoidal curve to obtain the mean densities of the liquid and vapor at the respective temperature. This approach has also been used by Muniz et al.^57^ and Bauer et al.^58^ Specifically, we used a hyperbolic tangent function to fit the densities of vapor and liquid at the upper and lower bounds of the liquid phase. With these liquid and vapor densities, we obtained the critical point by fitting according to
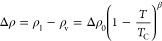
3
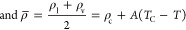
4which are called the universal scaling law of the coexistence densities and the law of rectilinear diameters, respectively.^59^ Like Muniz et al.,^57^ we used β = 0.326, which has been determined by Zinn-Justin.^60^

#### Canonical Simulations of Liquid and Vapor

Based on the results of the coexistence simulations, we performed further simulations in the canonical ensemble of pure liquid or pure vapor, respectively. From these simulations, we estimated the transition thermodynamics (see below) following the procedure as introduced by Muniz et al.^57^ In summary, at all temperatures, we simulated pure vapor and pure liquid in simulations with a constant box size and chose the box size in accordance with the respective densities from the coexistence simulations. For the simulation of the liquid, 512 water molecules were used, whereas only 128 water molecules were used for the vapor simulations. This saves the computation time, since the vapor exhibits very low (coexistence) densities, especially at low temperatures. To increase the efficiency, we also increased the cutoff distances for the simulations with low densities and avoided wasting computational effort on large numbers of sparsely populated cutoff boxes.

#### Isobaric–Isothermal Simulations

To estimate the density of the various water models in the liquid phase at various temperatures, we performed simulations in an isobaric–isothermal ensemble (*NPT*). Prior to these simulations, we minimized the initial structure. Furthermore, we performed a short equilibration at the respective temperatures in the canonical ensemble. After a short equilibration of the density in an initial *NPT* simulation, production runs of 10 ns were performed. All *NPT* simulations were performed at a pressure of 1 bar.

To quantify the temperature of spontaneous evaporation, we investigated the equilibrium density of the water models at elevated temperatures. Thus, we performed *NPT* simulations at high temperatures and determined the temperature at which the simulation box explosively expands, transitioning from the liquid to the vapor phase. To this end, two different approaches were evaluated. First, after equilibration of the temperature (in the canonical ensemble at a density corresponding to 300 K), we immediately started an *NPT* simulation at the respective high temperature. Therefore, the initial density was far off the equilibrium density. Second, we equilibrated the density stepwise; that is, an equilibrated structure of the next lower-temperature was used as the initial configuration for the next higher simulation. In the discussion, we refer to these approaches as pre-equilibrated density or not pre-equilibrated density.

Furthermore, we examined the effect of various parameters in the simulation setup on the apparent temperature of spontaneous evaporation. First, we tried out different heating rates, i.e., how long we simulated per temperature level. Besides that, we tested three different pressure coupling algorithms: the Berendsen algorithm,^61^ stochastic cell-rescaling (C-rescale),^62^ and the Parrinello–Rahman (PR) algorithm.^63^ Moreover, we investigated the influence of the system size on the apparent temperature of evaporation.

Apart from the temperature of spontaneous evaporation, we also estimated the temperature of the highest density. To this end, we performed *NPT* simulations of all water models at low temperatures. Subsequently, we quantified the mean density at the respective temperatures. The protocol for these simulations was the same as that for the *NPT* simulations at high temperatures. We interpolated these mean densities by fitting with the empirical function of the density of liquid water at different temperatures, according to Jones and Harris.^64^ We estimated the uncertainty of these properties—the highest measured density and the temperature at which we measured this density—by trajectory splitting. To this end, we simulated for a total simulation time of 30 ns, split this trajectory in six parts of 5 ns, discarded the first split, and evaluated the resulting five splits.

### Evaporation Thermodynamics

The enthalpy of evaporation, Δ*H*, was estimated at different temperatures for all water models according to
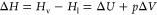
5
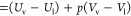
6

To this end, we calculated the mean internal energy from the respective canonical simulations of pure vapor and liquid, *U*_v_ and *U*_l_. Furthermore, from the simulations of pure vapor, we estimated the corresponding saturation vapor pressure, *p*. Again, we followed the approach outlined by Muniz et al.^57^

## Results and Discussion

Comparative liquid–vapor coexistence simulations and *NPT* simulations were performed on 11 different water models. First, we present and discuss the results from the liquid–vapor coexistence simulations followed by the *NPT* simulations. This section is concluded by showing and discussing correlations between the obtained points in phase space and the properties of the water models.

### Liquid–Vapor Coexistence

**Figure 2:**
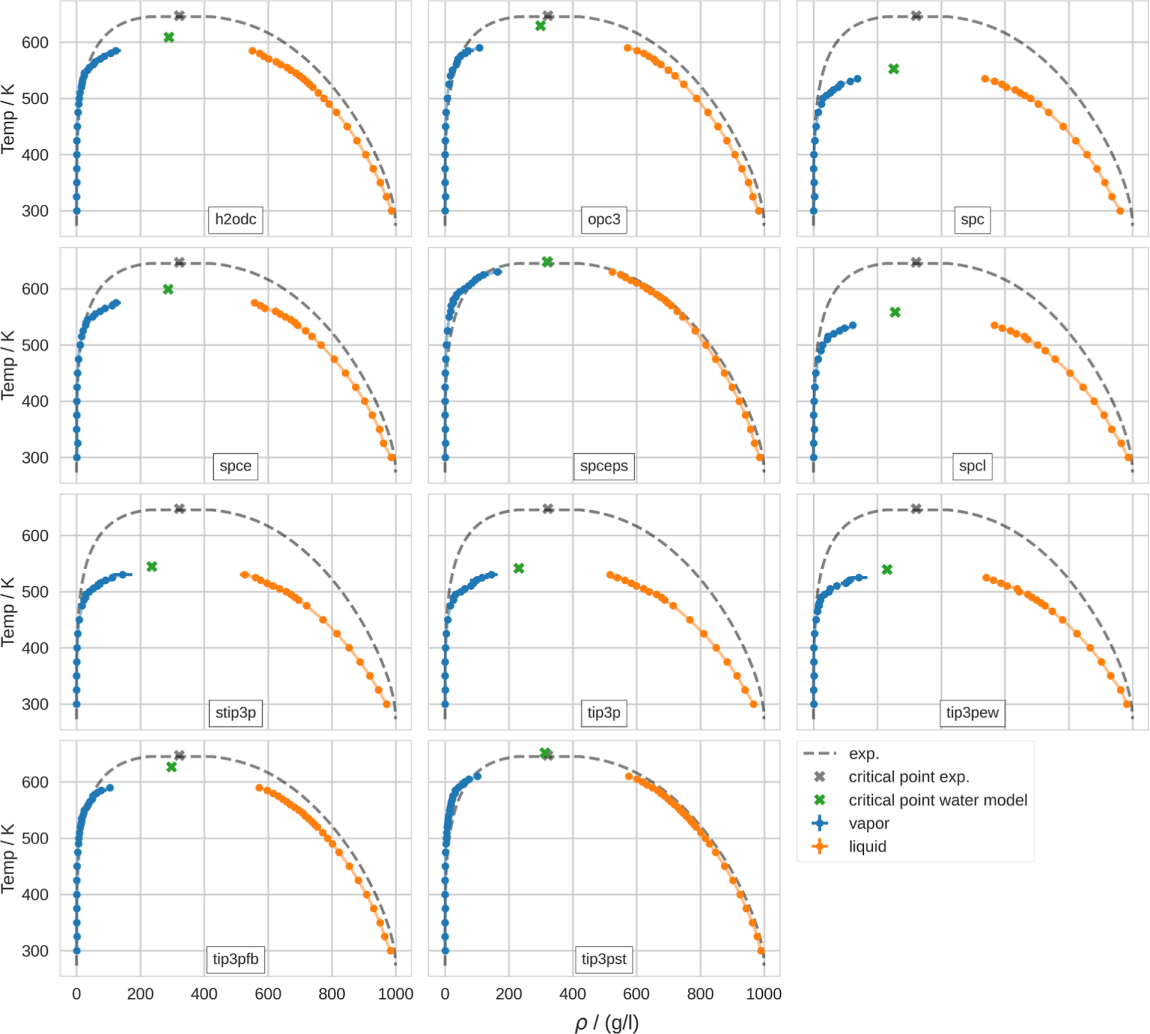
Liquid–vapor coexistence curves of different water models. The results for the different water models are shown in separate panels. The water models are indicated in the respective text boxes. The densities of the vapor are indicated in blue circles and the densities of the liquid at the respective temperatures are indicated in orange circles. An estimation for the critical point of the respective model is given as a green cross. The coexistence curve of water in experiments is plotted as a dashed line, including the critical point of water in experiments plotted as a gray cross. Uncertainties have been estimated from trajectory splitting.

We obtained liquid–vapor coexistence density curves for all water models, which are visualized in Figure 2. There, the comparison to the experimental curve is shown, which strongly varies between water models. We found that it is particularly challenging to obtain accurate estimates of the coexistence densities at temperatures close to *T*_C_. This stems from the fact that it is increasingly difficult to center the liquid slab in the simulation box at all frames, because liquid and vapor phases are difficult to distinguish as the difference in density gets smaller. However, this centering is crucial to obtain a clean sigmoidal fit of the coexistence densities. In these coexistence density diagrams, it appears that—up to a certain temperature—many water models seem to reproduce the vapor density better than the liquid density. However, the relative difference in densities for the vapor phase is not well visible at this scale. As a rule, water models that reproduce the density of the liquid phase well over a wide range of temperatures (for example, SPC/ϵ and TIP3P-ST) also yield good accuracy for the critical point. We plot and further discuss the obtained critical points below. Interestingly, the seemingly best-performing water models (SPC/ϵ and TIP3P-ST) apparently underestimate the vapor density above 550 K.

#### Critical Point

We obtained the critical points for the different water models by fitting the liquid–vapor coexistence curves. Like Muniz et al.,^57^ we performed a sigmoidal fit to this end (see above). Our estimations are also marked in Figure 2, and the respective values are listed in Table 2.

**Table 2:** Characteristic Points in the Phase Diagram of the Here-Used Water Models: Critical Point, Point of Maximum Density, and Temperature of Spontaneous Evaporation at *p* = 1 bar^a^

	*T*_C_	ρ_C_	*T*_MD_	ρ_MD_	*T*_evap_
H2ODC	608.6 ± 2.0	289.1 ± 2.9	254.4 ± 1.1	1008.3 ± 0.3	581.3 ± 0.7
OPC3	629.1 ± 2.1	299.0 ± 2.1	253.9 ± 1.1	1006.8 ± 0.2	593.7 ± 1.2
SPC	552.3 ± 0.8	251.5 ± 2.5	225.0 ± 0.9	1009.5 ± 0.3	531.5 ± 1.5
SPC/E	599.1 ± 1.3	287.2 ± 1.8	249.8 ± 0.5	1013.1 ± 0.3	573.5 ± 1.4
SPC/ϵ	648.3 ± 1.1	319.9 ± 1.9	270.4 ± 1.0	1001.2 ± 0.2	620.5 ± 1.3
SPC/L	558.3 ± 1.1	256.2 ± 2.6	220.7 ± 1.4	1037.9 ± 0.2	535.7 ± 1.7
sTIP3P	544.4 ± 1.4	235.5 ± 2.6	202.0 ± 0.8	1042.5 ± 0.7	526.5 ± 1.7
TIP3P	541.6 ± 2.5	231.6 ± 3.2	199.1 ± 2.0	1039.2 ± 0.6	524.1 ± 1.0
TIP3P-EW	539.1 ± 0.9	230.8 ± 4.1	222.2 ± 1.2	1035.5 ± 0.4	520.1 ± 1.6
TIP3P-FB	626.8 ± 1.2	297.6 ± 1.2	258.5 ± 1.5	1004.2 ± 0.2	592.9 ± 1.9
TIP3P-ST	651.7 ± 2.6	312.9 ± 1.2	277.8 ± 2.2	1000.2 ± 0.2	609.5 ± 1.1
Exp	647.1	322	276.2	999.1	

a All temperatures, *T*, are given in [K], and all densities, ρ, are given in [g/l]. The values for *T*_evap_ have been estimated with the C-rescale pressure coupling algorithm. Uncertainties for the critical point and for the point of maximum density have been obtained from trajectory splitting; the uncertainties for *T*_evap_ have been obtained from repeated execution of the heating simulations. We give experimental references in the bottom row, where available.^65^

**Figure 3:**
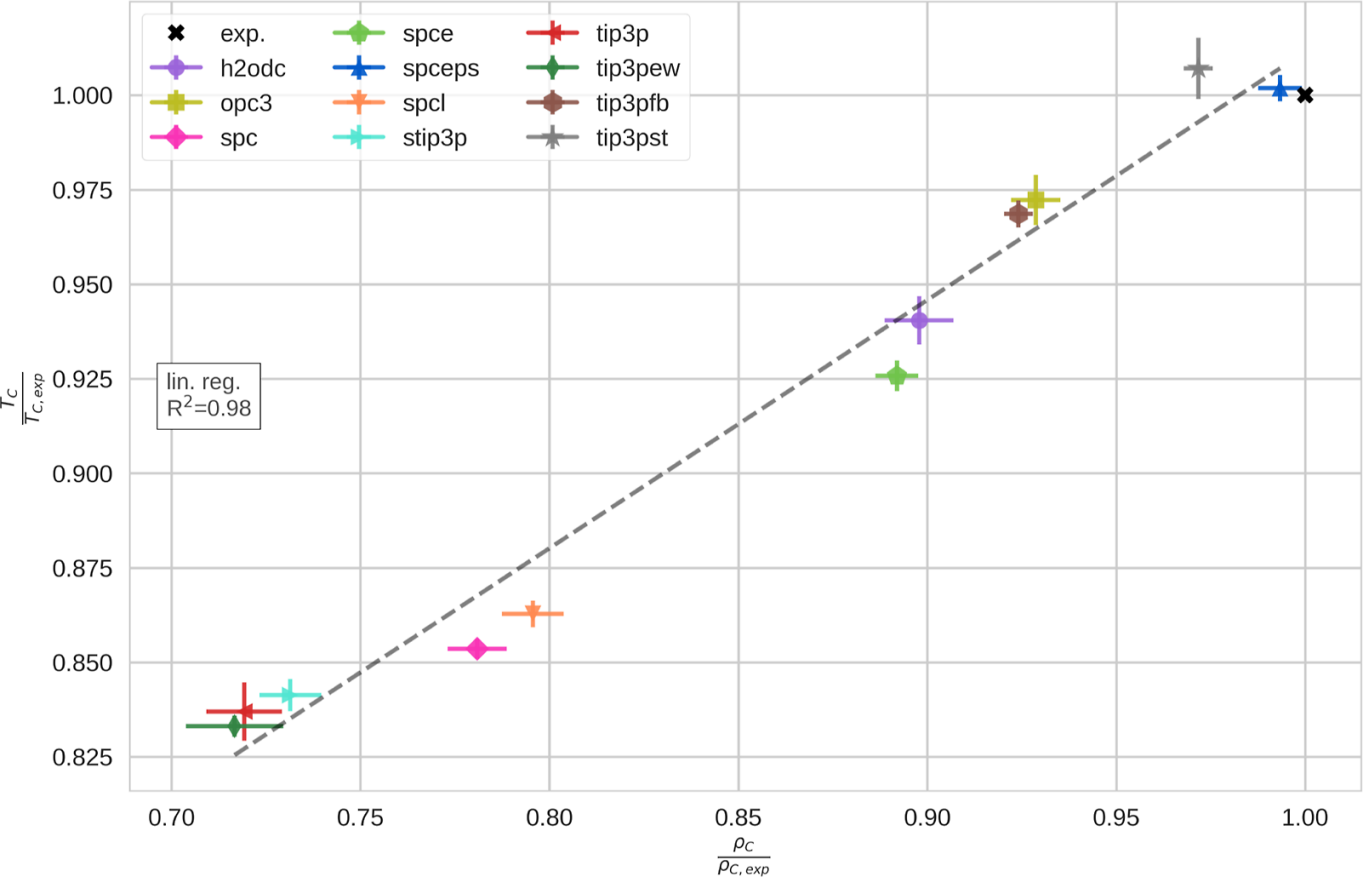
Critical points of different water models. We scaled the respective values for the critical temperature and the critical density by the experimental reference value. Thus, relative critical temperatures and densities, respectively, are compared. The different water models are indicated by different symbols in different colors, including the experimental reference as a black cross.^65^ Uncertainties have been estimated by trajectory splitting.

For comparison with the experimental values, we visualize the critical points obtained with all water models as a scatter plot, as shown in Figure 3. Generally, we notice that modern water models reproduce the critical point significantly better than the less modern water models. An exception to this trend is SPC/E, which is considerably old, but performs almost as good as modern water models. According to our data, in comparison to all the water models tested here, SPC/ϵ reproduces the critical point best.

We notice a significant linear correlation (*R*^2^ = 0.98) between the critical temperatures and the critical densities obtained with different water models. Accordingly, it should be assumed that it is not possible to optimize the critical density independent of the critical temperature. We assume that this linear correlation might to some extend be imposed by our fitting procedure: We applied the law of rectilinear diameters, but alternative (nonlinear) scaling laws would probably yield even more accurate predictions of the critical point of water.^66^ Also, like Muniz et al.,^57^ we applied the universal scaling law with the critical exponent, as determined by ref (60). Potentially, given the extent of our data, we might have considered optimizing the critical exponent. However, given the consistency of the obtained coexistence densities, we rate the error due to the choice of scaling laws to be small.

We find that all here-used water models exhibit a critical density lower than that observed in experiment. Equally, almost all water models underestimate the critical temperature. Only TIP3P-ST shows a slightly higher critical temperature (thereby, slightly deviating from the linear correlation line of *T*_C_ vs ρ_C_, as discussed above). While SPC/ϵ also slightly overestimates the critical temperature, this deviation is within the uncertainty of our estimates.

Other authors have investigated the critical point of selected three-point water models in computational studies before.^67−70^ In comparison to other studies, we generally report good qualitative agreement. However, we notice that the cited studies report critical temperatures slightly higher than those found in our study. We believe that this is potentially because these studies did not include simulations as close to the critical point as we did. Possibly, the reason for this choice of simulation temperatures is that the distinction between liquid and vapor densities becomes increasingly difficult. Note also that we simulated longer than previous studies and with a higher temperature resolution. Clearly, the amount of sampling we invested here was not feasible 7–17 years ago.

#### Evaporation Thermodynamics

**Figure 4:**
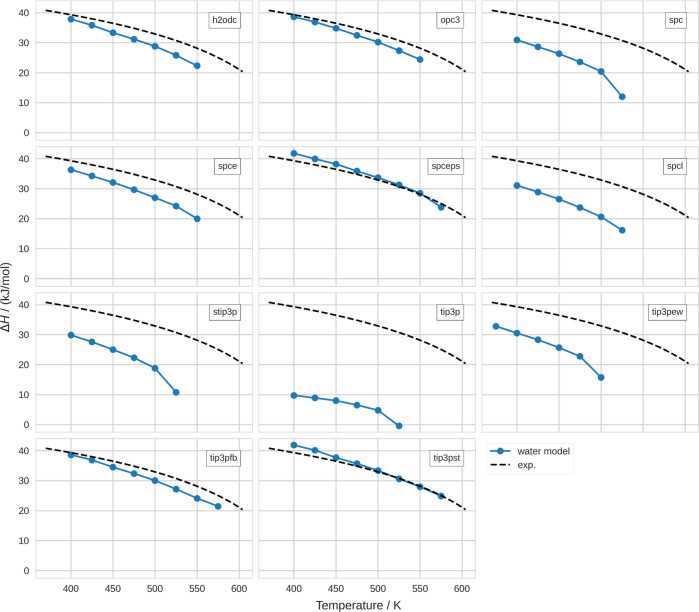
Enthalpy of evaporation, Δ*H*. In the different panels, the results of the respective water models (blue circles) in comparison to the experimental reference (black dashed line) are indicated.

From canonical simulations of pure vapor and liquid, we obtained the transition thermodynamics. We calculated the enthalpy of evaporation, Δ*H*, at various temperatures for the difference in density and internal energy in both phases, as outlined in the Materials and Methods section. We show Δ*H* values for different water models in Figure 4. It can be noticed that most older water models—namely, SPC, SPC/E, SPC/L, sTIP3P, TIP3P, and TIP3P-EW—show significant deviation from the experimental reference. Among those, SPC/E performs the best. In comparison, the modern water models perform significantly better.

Furthermore, comparing these results with our results for the critical point, it appears that the reproduction of the critical temperature seems to follow the same trend: water models with a particularly low critical temperature show a particularly low Δ*H*. Analogously, water models with a particularly high critical temperature show a particularly high Δ*H*. This is not surprising as these quantities are thermodynamically linked.^71^ We find that SPC/ϵ and TIP3P-ST are the only two water models that exhibit Δ*H* values above the experimental curve at some temperatures. This is consistent with our finding that these water models show an underestimation of the density in the vapor phase.

We may further split up the enthalpy into internal energy and volume work, Δ*H* = Δ*U* + *p*Δ*V*. However, we found that the enthalpy of the liquid–vapor transition is governed by the internal energy. In comparison, the volume work is 1 order of magnitude smaller. This is consistent for all water models and also with experiment. We show the respective figures in the Supporting Information Section C.

### Isobaric–Isothermal Simulations

Here, we present all of the results related to simulations in the *NPT* ensemble. This section is subdivided into the results obtained at high simulation temperatures, which yielded the temperature of spontaneous evaporation, and the results at low temperatures, which yielded the point of maximum density. All *NPT* simulations of the different water models at various temperatures were performed at a pressure of 1 bar.

#### Spontaneous Evaporation

We estimated the temperature at which the different water models spontaneously evaporate, *T*_evap_, from *NPT* simulations at high temperatures, as given in Table 2. Generally, we found that there are significant differences between water models: the values span a range between roughly 520 and 620 K. The influence of various simulation parameters on *T*_evap_ is discussed below.

##### Influence of System Size

We did not find any significant dependence of *T*_evap_ on the system size, in the range of system sizes that were studied: We investigated systems with 216 up to 1728 water molecules (in steps of 216). Presumably, the results may actually deviate for even smaller systems. However, nowadays smaller systems are hardly ever simulated any more. Even with highly complex and computationally demanding models of water—such as seven-point water models, or flexible and polarizable models—systems sizes of 216 molecules may easily be simulated for multiple nanoseconds within a reasonable amount of time.^57^ Yet, we want to note that we expect the apparent transition temperature to depend on the fluctuations of the density. Since the magnitude of fluctuations depends on the number of particles, simulations with larger systems may presumably yield estimations of *T*_evap_ with lower variance.

##### Influence of the Pressure-Coupling Algorithm

We found that *T*_evap_ depends on the used pressure coupling algorithm. Generally, the results with the different algorithms correlated well and may be related to each other with an offset. We found that *T*_evap_ obtained with the Berendsen pressure coupling algorithm is generally ∼20 K higher than the values obtained with C-rescale. Furthermore, the PR pressure coupling algorithm yields values that are another ∼10 K lower than C-rescale. In Table 2, we show the results obtained with the C-rescale algorithm.

Generally, to investigate such transition, we would recommend relying on the C-rescale algorithm. The Berendsen barostat is known not to reproduce the fluctuations of the box volume correctly,^62^ which is probably important for spontaneous evaporation. Also, the algorithms by Berendsen—both, the pressure-coupling, but also the analogous implementation for temperature-coupling—do not sample the proper thermodynamic ensemble.^72−75^ On the other hand, the PR algorithm should actually not be used for the equilibration of the density: if the initial condition of the system is far from the equilibrium value, this algorithm will overshoot the equilibrium density distribution (depending on how far off the density was in the first place). This would potentially lead to *early evaporation* (i.e., at lower temperatures). Thus, we expect the C-rescale algorithm to be the most reliable for such a study.

##### Influence of Pre-Equilibration of the Density

We found that it has an influence on the apparent temperature of spontaneous evaporation, whether the density was pre-equilibrated or not. Naively generated initial simulation boxes (corresponding to the density at 300 K) may evaporate in *NPT* simulations at even lower temperatures if directly simulated at high temperatures (without pre-equilibrated density). We found that evaporation may happen at temperatures ∼5–10 K lower in this scenario. We investigated this effect only with the Berendsen pressure-coupling algorithm, but we expect that similar shifts may be observed with the C-rescale algorithm. With the PR algorithm, this effect may potentially be even stronger since this algorithm overshoots the equilibrium density, if the initial density is far off (see above). As a consequence, we generally recommend to pre-equilibrate the density at an intermediate temperature, if simulations at high temperatures, close to *T*_evap_, are desired.

##### Influence of the Heating Rate

For fast heating rates, we expect to see a hysteresis of spontaneous evaporation: in very short simulations (per temperature step), spontaneous evaporation may not occur. Therefore, fast heating rates may lead to a biased result for *T*_evap_. In order to quantify a sufficiently slow heating rate, we performed simulations with various simulation times per temperature. Representatively, we chose SPC/ϵ for this study. We found a convergence of the measured transition temperature with 5 ns per temperature step with simulations of 216 water molecules. This corresponds to an average heating rate of 0.2 K/ns. The assessment of this convergence is shown in the Supporting Information Section F.

##### Spontaneous Evaporation vs Boiling

We emphasize that *T*_evap_ is generally not equal to the boiling temperature, *T*_B_. Therefore, our results should not be compared with the experimental boiling temperature. The boiling point is defined as the temperature at which “*the vapor pressure of the liquid equals the environmental pressure surrounding the liquid*”.^76^ Thus, boiling describes evaporation under certain specific conditions, which are further discussed below. Generally, it is important to notice that both these characteristic temperatures—*T*_evap_ and *T*_B_—generally depend on the pressure and on the eventual salt concentration.^77^

The process of boiling is often characterized by the formation of bubbles in a liquid during heating: under continuous transfer of heat (e.g., on a stove) during boiling, a (more or less) stable temperature may be measured, which is referred to as the boiling temperature. For the growth of bubbles, the vapor pressure within the bubbles must exceed the pressure at the bubble surface, which includes surface tension and the ambient pressure. In contrast, in our simulations, we do not model such heat transfer, and also, no bubbles form. Thus, spontaneous evaporation in our simulations occurs above the boiling point in the regime of superheated liquids. In this region of the phase diagram, the liquid phase is metastable. Analogous behavior is seen for the condensation behavior, where a metastable vapor phase exists at considerably lower temperatures. Therefore, what we measure in our simulation is the temperature at which the metastable (superheated) liquid transitions to the vapor phase (at a given pressure). Other authors have called this behavior, for example, rapid evaporation at the superheated limit,^78^ explosive boiling,^79−81^ or phase explosion.^82^ Generally, boiling has been successfully simulated, however, in complex setups.^81,83^ While we would generally be very interested in estimating the actual boiling point of the water models studied here, it is out of the scope of this study. Experimental studies on superheated water exist. Temperatures of 473–510 K^84^ or even 518 K^85^ have been measured. The referenced experiments on superheated water were done with droplets in hot jets of water, which is questionable to compare with, as these droplets are probably very unstable and short-lived (presumably in nonequilibrium). Also, the eventual thermodynamic conditions (such as the density and pressure in these droplets) are challenging to determine. As a result, we do not have any experimental reference value for *T*_evap_.

#### Temperature of the Highest Density

We obtained the densities of the different water models in the liquid phase at low temperatures (see Supporting Information Section D). From the densities, one can identify the point in the phase diagram where the density is maximal. In Table 2, we compare the highest density, ρ_MD_, and the temperature of highest density *T*_MD_ with experimental data.

**Figure 5:**
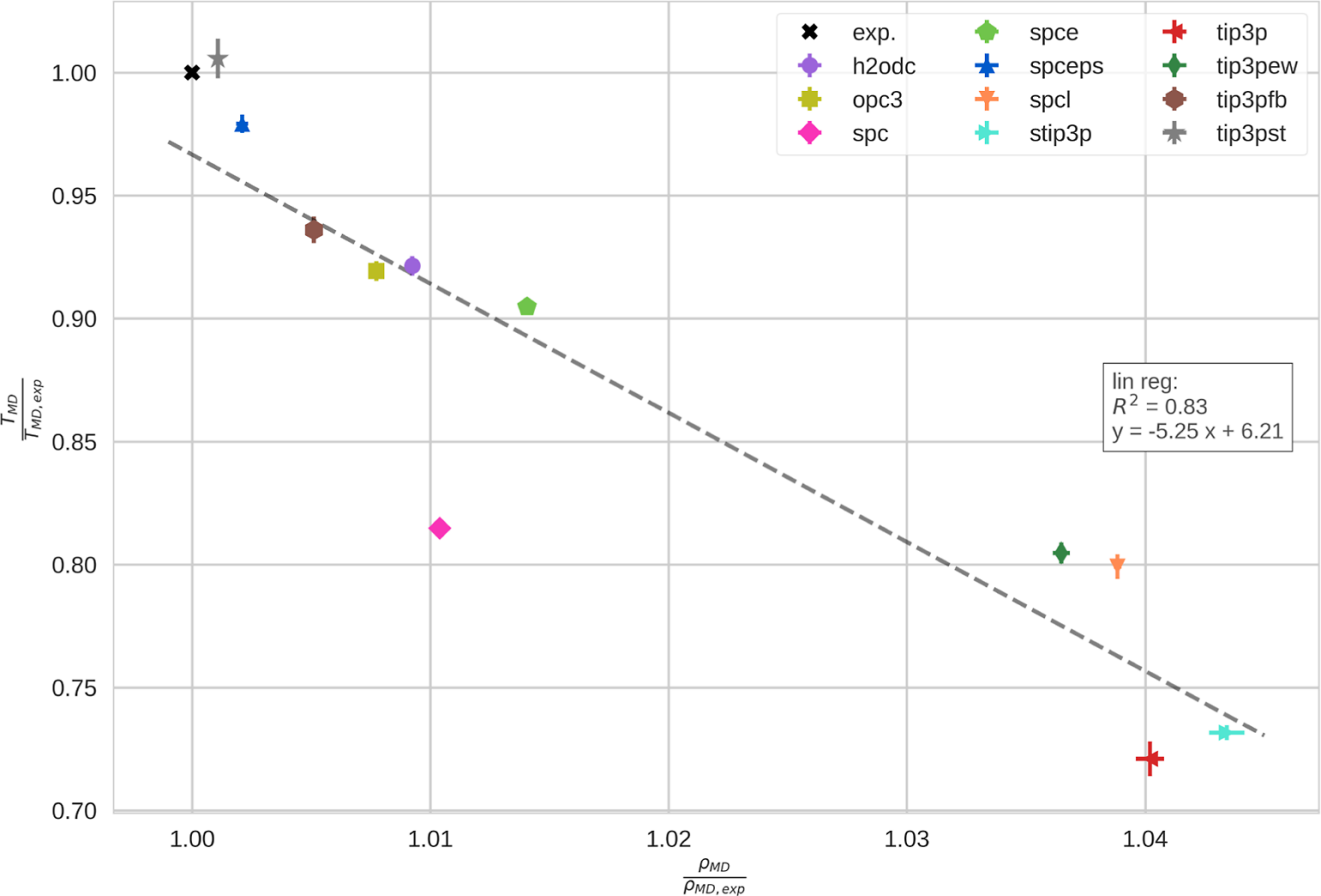
Temperature of highest density. Here, we show the highest density of the respective water models, ρ_MD_, and the temperature at which it occurs, *T*_MD_. We rescaled the values obtained with the respective water models by the experimental reference values. The results with the various water models are indicated as symbols in different colors. We determined uncertainties from trajectory splitting. The experimental reference is shown as a black cross.^65^

Furthermore, in Figure 5, we visualize the relative values with respect to the experimental value. Generally, we notice that all water models overestimate ρ_MD_. Furthermore, almost all models underestimate *T*_MD_, with TIP3P-ST being the only exception. We notice that the relative deviations in ρ_MD_ are small, generally below 5%. In comparison, the relative differences in terms of *T*_MD_ are larger, reaching more than 25% in exceptional cases. Generally, there is a clear trend for newer water models to reproduce this point in the phase diagram better than older models. Comparing the results for the critical point with the results for the point of maximum density, we find very similar trends.

### Correlation between Different Points in the Phase Diagram

To evaluate the consistency of the reproduction of the phase diagrams for the different water models, we compared the different characteristic temperatures. Thus, we looked at the correlations, e.g., of *T*_evap_ and *T*_C_, obtained with the various water models, respectively. We found that *T*_evap_ is on average roughly 37 K below *T*_C_. This relation between the two temperatures may be described slightly better by a full linear fit; however, only fitting an offset already yields very good correlation, *R*^2^ ≃ 0.96. We show this correlation in the Supporting Information Section F. Hence, we expect *T*_evap_ ≃ *T*_C_ – 37 K to be a valid estimate for the temperature of spontaneous evaporation of rigid three-point water models (if the density was pre-equilibrated). This relation was determined with the C-rescale pressure-coupling algorithm, but analogous rules of thumb may be formulated for other pressure-coupling algorithms (see above). We will validate this estimation for four-point water models in the future.

Furthermore, we compared *T*_MD_ and *T*_C_ and found that *T*_C_ is on average roughly 375 K higher. For reference, in experiment, this difference is 371 K. However, the relation between these two temperatures may be described significantly better by a full linear fit. Nevertheless, for water models that show low *T*_MD_, one should also expect low *T*_C_. Lastly, we also found good correlation between *T*_MD_ and *T*_evap_. The corresponding figures for both these relations are shown in Supporting Information Section E.

### Correlation between the Evaporation Temperature and Model Parameters

To eventually understand better what determines the evaporation behavior of three-point water models, we investigated the correlations between model parameters and the apparent temperature of spontaneous evaporation. Indeed, the relation between the model parameters and *T*_evap_ is presumably complex. Generally, we expected stronger interactions between molecules to stabilize the liquid phase. However, the strength of water–water interactions depends on electrostatic and LJ interactions. Thus, depending on the water model, weaker electrostatics may potentially be compensated by stronger LJ interactions and vice versa. A decisive difference between these two interaction types is the symmetry: the dipolar (or rather multipolar) arrangement of the charges leads to complex spatial arrangements, to hydrogen bond networks, and so on. In contrast, the LJ interactions in the force field are modeled as uniform spherically symmetric contribution, because three-point water models commonly only have one LJ-interaction site (with the exception of sTIP3P).

Our first approach was separately correlating all of the model parameters of the various water models with *T*_evap_. We show the corresponding figures in Supporting Information Section F. For all parameters, we found only a weak correlation. We recognize a weak trend that a small ϕ_HOH_ seems to lead to low *T*_evap_, with SPC being the outlier from this trend. Similarly, we find a weak trend between σ_O_ and *T*_evap_. The biggest outlier from this trend was TIP3P-EW.

**Figure 6:**
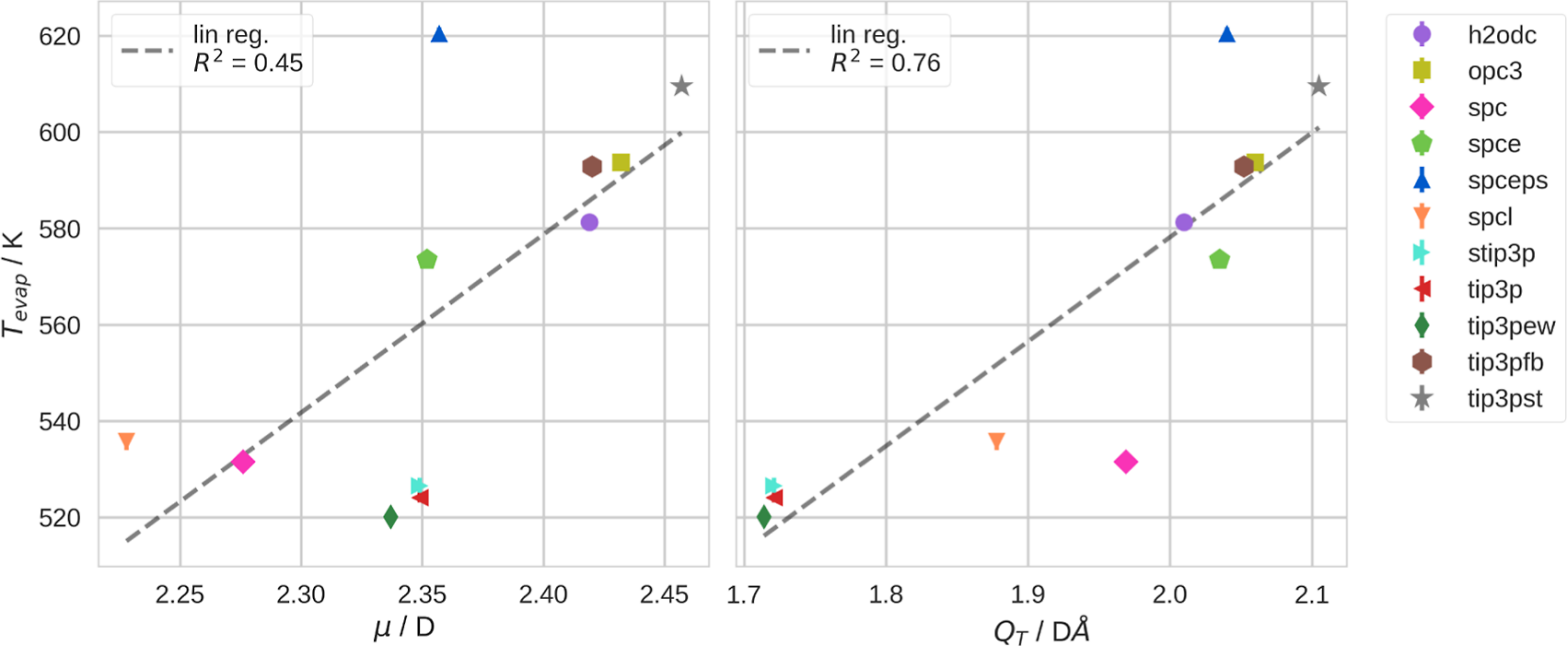
Correlation between the dipole moment, μ, and the quadrupole moment, *Q*_T_, with the temperature of spontaneous evaporation, *T*_evap_. The different water models are indicated as colored spheres and a linear fitting dashed black line.

Furthermore, we investigated the correlations between dipole moment, μ, and the quadrupole moment, *Q*_T_, with *T*_evap_ (see Figure 6). We notice some weak proportional correlations between μ and *T*_evap_. However, the correlation between *Q*_T_ and *T*_evap_ is significantly stronger. Accordingly, we hypothesize that the spatial arrangement and the strength of the partial charges of water seem to be important for reproducing the liquid–vapor transition. This is not surprising as it has already been shown and discussed that *Q*_T_ is important for the reproduction of other parts of the phase diagram of water.^50^ In this context, we also want to mention that Gubskaya and Kusalik^86^ have found that the dipole moment of liquid water might actually be weakly temperature-dependent. Accordingly, the quadrupole moment is probably (to some extent) temperature-dependent, too. Unfortunately, rigid nonpolarizable water models do not allow for such temperature-dependent adaptations without reparameterization.

We notice that SPC/ϵ shows particularly large deviations from this trend. This deviation may be explained by the particularly strong LJ interactions of this model. From this data point, we reason that while *Q*_T_ seems to be of particular importance, the LJ interactions are clearly also relevant for the thermodynamics of this transition. This hypothesis is also in line with the results for Δ*H*, where SPC/ϵ exhibits particularly high values. Furthermore, we notice that SPC and SPC/L, the two water models with the lowest partial charges in our set, lie noticeably below the trend line. This is a hint that a particular value of *Q*_T_ may be obtained with low charges, if compensated with large *d*_OH_ and large ϕ_HOH_. However, low partial charges lead to weak hydrogen bonds and thus reduced water–water interactions. Accordingly, *Q*_T_ alone does not yield a standalone prediction of the evaporation temperature.

## Conclusions

Here, we investigated the critical point, the temperature of spontaneous evaporation, and the point of highest density of various rigid three-point water models. These water models were mostly designed to be used under approximately ambient conditions, i.e., around 1 bar and at room temperature. Thus, the performance at high temperatures is not guaranteed. Nevertheless, we found that modern three-point water models generally perform quite decent at high temperatures, better than old water models. This finding is also in line with those of previous studies.^34^ In particular, we found that modern three-point water models reproduce the critical point surprisingly well.

Potentially, more complex models, e.g., four-point water models, may perform even better at high temperatures. Indeed, there is evidence that—for perfect accuracy over a wide range of temperatures—flexible and polarizable models may yield even higher accuracy, since there is evidence that temperature-dependent polarization effects may be relevant.^57,69,87−89^ Besides that, also ab initio models may potentially perform better at modeling liquid–vapor coexistence (depending on the QM model that is used for these simulations).^26,90−92^ Nevertheless, we found that modern three-point water models show decent agreement with experiments, also at high temperatures. Thus, taking into account the common trade-off between accuracy and performance, these models might be of sufficient accuracy at elevated temperatures (depending on the particular application, of course).

Lastly, we found that the quadrupole moment of the water models shows a significant correlation with the evaporation temperature of the water models. This is additional evidence for the connection between the quadrupole moment of water, which encodes geometry and charge distribution, and the phase diagram. However, we found that particularly strong LJ interactions may disturb this correlation. Also, particularly small partial charges may cause deviations, even if the tetrahedral quadrupole moment is not too much affected. In sum, we conclude that geometry and partial charges are of major importance, but also LJ interactions need to be parameterized accordingly to further improve the accuracy of common water models for simulations at elevated temperatures.

## Supporting Information Available

(A) Investigated water models, (B) ranking of LJ parameters, (C) evaporation thermodynamics, (D) temperature of highest density, (E) correlations, and (F) convergence of estimations (PDF)
