# Supplementary material for: Liquid–Vapor Coexistence and Spontaneous Evaporation at Atmospheric Pressure of Common Rigid Three-Point Water Models in Molecular Simulations
Source: J Phys Chem B. 2024 Mar 1;128(10):2457–68. doi: 10.1021/acs.jpcb.3c08183 (PMC10945489; doi:10.1021/acs.jpcb.3c08183)
Supplement: Supplementary file 1 — jp3c08183_si_001.pdf [file jp3c08183_si_001.pdf]

Supporting Information for Publication:

Liquid-Vapor Coexistence and Spontaneous Evaporation at Atmospheric Pressure of Common Rigid Three-Point Water Models in Molecular Simulations

Patrick K. Quoika[†,*] and Martin Zacharias[†]

[†] *Center for Functional Protein Assemblies, Technical University of Munich, Ernst-Otto-Fischer-Str. 8, Garching 85748, Germany*

E-mail: patrick.quoika@tum.de

Contents

Supporting Information **S3**

A Investigated Water Models S3

A.1 Dipole Moment and Quadrupole Moment S5

B Ranking of Lennard-Jones Parameters S6

C Evaporation Thermodynamics S7

C.1 Inner Energy S7

C.2 Volume Work S9

D Temperature of Highest Density S10

E Correlations S11

E.1 T_{evap} vs. T_C S11

E.2 T_C vs. T_{MD} S12

E.3 T_{evap} vs. T_{MD} S13

E.4 Correlation to Model Parameters S14

F Convergence of Estimated Values With Simulation Time S14

Supporting Information

Below we provide the supporting information for the manuscript:

> Liquid-Vapor Coexistence and Spontaneous Evaporation at Atmospheric Pressure of Common Rigid Three-Point Water Models in Molecular Simulations.

We structured this document in short subsections, as listed in the table of contents above and as mentioned in the main manuscript.

A Investigated Water Models

Here, we provide additional information about the used water models, which did not find space in the main manuscript. In particular, we summarize the parametrization procedure and where the abbreviations come from, eventually. The references for these water models can be found in the main manuscript.

H2ODC The authors parametrized a water model to reproduce the density, the enthalpy of vaporization (at T = 300 K) and the dielectric constant. Accordingly, **DC** stands for *dielectric corrected*. They used geometric features of both TIP3P and SPC as a template structure. In the same publication, they parametrized force field parameters for Chloromethanes. Furthermore, they also perform a reparametrization of SPC, called SPC/DC, there.

SPC **S**imple **P**oint **C**harge model. One of the earliest water models ever. We consider SPC and TIP3P to be the pioneer models in the field of MD simulations.

TIP3P **T**ransferable **i**ntermolecular **p**otential **3-p**oint. One of the earliest water models ever. We consinder SPC and TIP3P to be the pioneer models in the field of MD simulations.

sTIP3P The CHARMM variant of TIP3P. It features an additional weak repulsive interaction site on the hydrogen atoms. Besides that it is identical to regular TIP3P. Why the authors introduced this modification is not clear to us: In their publication of the CHARMM22, they cited the PhD Thesis of W.E. Reiher, III. from 1985, which is not accessible for us.

SPC/E This is the **extended** simple point charge model. It is a reparametrization of SPC to include a correction for the self-energy term in the effective pair potential.

TIP3P-EW Reparametrization of the TIP3P model. The authors optimized the model to be used with the particle mesh **ew**ald method.

TIP3P-FB The authors reparametrized the TIP3P model according to the principle of **f**orce **b**alance (FB). Generally, the Force Balance method was developed to automatically derive accurate force field parameters for various molecules. In the same publication the authors also present TIP4P-FB.

SPC/ϵ The authors reparametrized the SPC model in order to reproduce the dielectric constant, ϵ, and the temperature of maximum density better. To this end, they varied the partial charges and adapted the LJ parameters accordingly. In the process, they left the geometry unchanged.

SPC-L The authors do not specifically explain the L in the name, but we believe it stems for the fact that it is **l**onger than SPC or SPC/E. Generally, this is the water model with the longest bond length in our set. This model was the result of a systematic parameter screening, where the authors investigated the possibility of simultaneous optimization of the properties of SPC-type water models. This water model has been intended to be used with the GROMOS force field.

OPC3 **O**ptimized **p**oint **c**harge **3**-point water model. Parametrized in an equivalent way to OPC, which is a 4-point water model. In ther parametrization, the authors focused

on the reproduction of the multipole moments of water.

TIP3P-ST The authors used the Force-Balance optimization procedure to reparametrize TIP3P to reproduce the **s**urface **t**ension (ST) better. In the same paper, they also reparametrized the 4-point analogue of TIP3P (TIP4P), yielding TIP4P-ST.

A.1 Dipole Moment and Quadrupole Moment

In our analysis, we investigated the correlation of dipole moment, μ and (tetrahedral) quadrupole moment, Q_T, with the critical temperature. Thus, we list μ and Q_T of the here-used water models in Table S1.

Table S1: Dipole moment, μ, and tetrahedral quadrupole moment, Q_T, of the here-used water models. μ is given in [D], Q_T in [DÅ].

	μ	Q_T
H2ODC	2.419	2.01
OPC3	2.432	2.06
SPC	2.276	1.97
SPC/E	2.352	2.04
SPC/ϵ	2.357	2.04
SPC/L	2.228	1.88
sTIP3P	2.349	1.72
TIP3P	2.349	1.72
TIP3P-EW	2.337	1.71
TIP3P-FB	2.420	2.05
TIP3P-ST	2.457	2.10

B Ranking of Lennard-Jones Parameters

Below, we visualize the Lennard-Jones (LJ) parameters of the here-used water models. In each sub figure, we ordered the water models according to the respective parameters.

We notice that there is one particular outlier in the distribution of ϵ_O, i.e., TIP3P-EW. Besides that, we notice that five out of eleven water models have been parametrized with 0.15 < ϵ_O < 0.16 kJ/mol. There is no clear trend that newer water models have higher ϵ_O: On the one hand, SPC/ϵ (2015), OPC3 (2016) are above the median value; on the other hand, TIP3P-ST (2019) is significantly below and TIP3P-FB (2014) is almost equal to the median value.

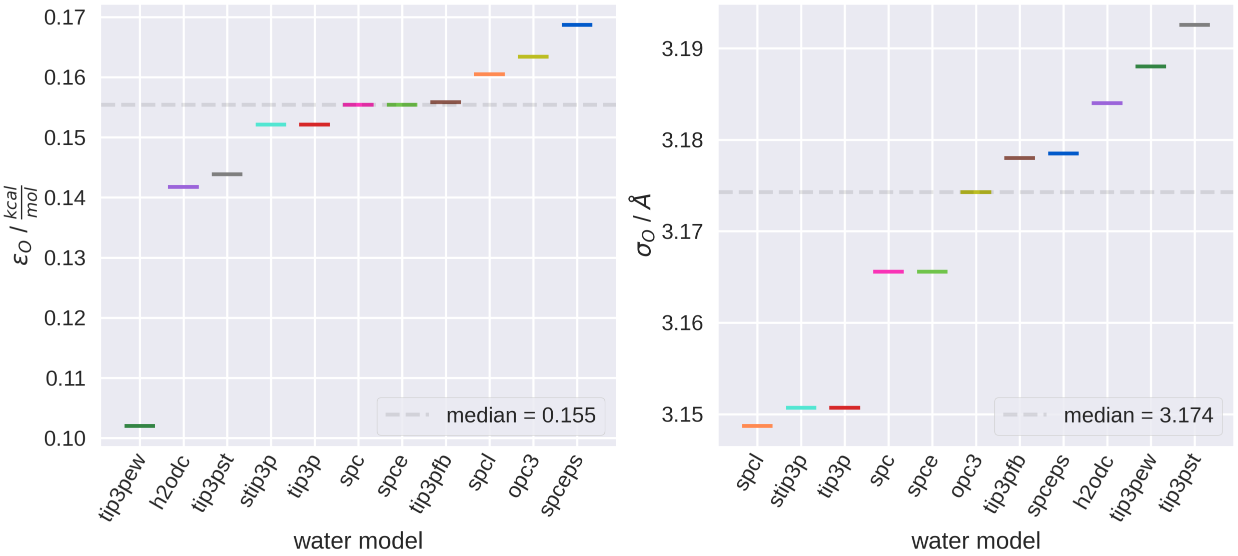

Figure S1: Ranking of the LJ-interaction parameters of the here-used water models.

C Evaporation Thermodynamics

In the main manuscript we show the enthalpy of evaporation, ΔH, at different temperatures for various water models. ΔH may be further split up into inner energy and volume work. Below, we show these two contributions separately.

C.1 Inner Energy

Below, we show our estimation of the difference in inner energy of vapor and liquid phase at different temperatures for different water models. These values have been obtained from separate canonical simulations of vapor and liquid phase at the respective temperature.

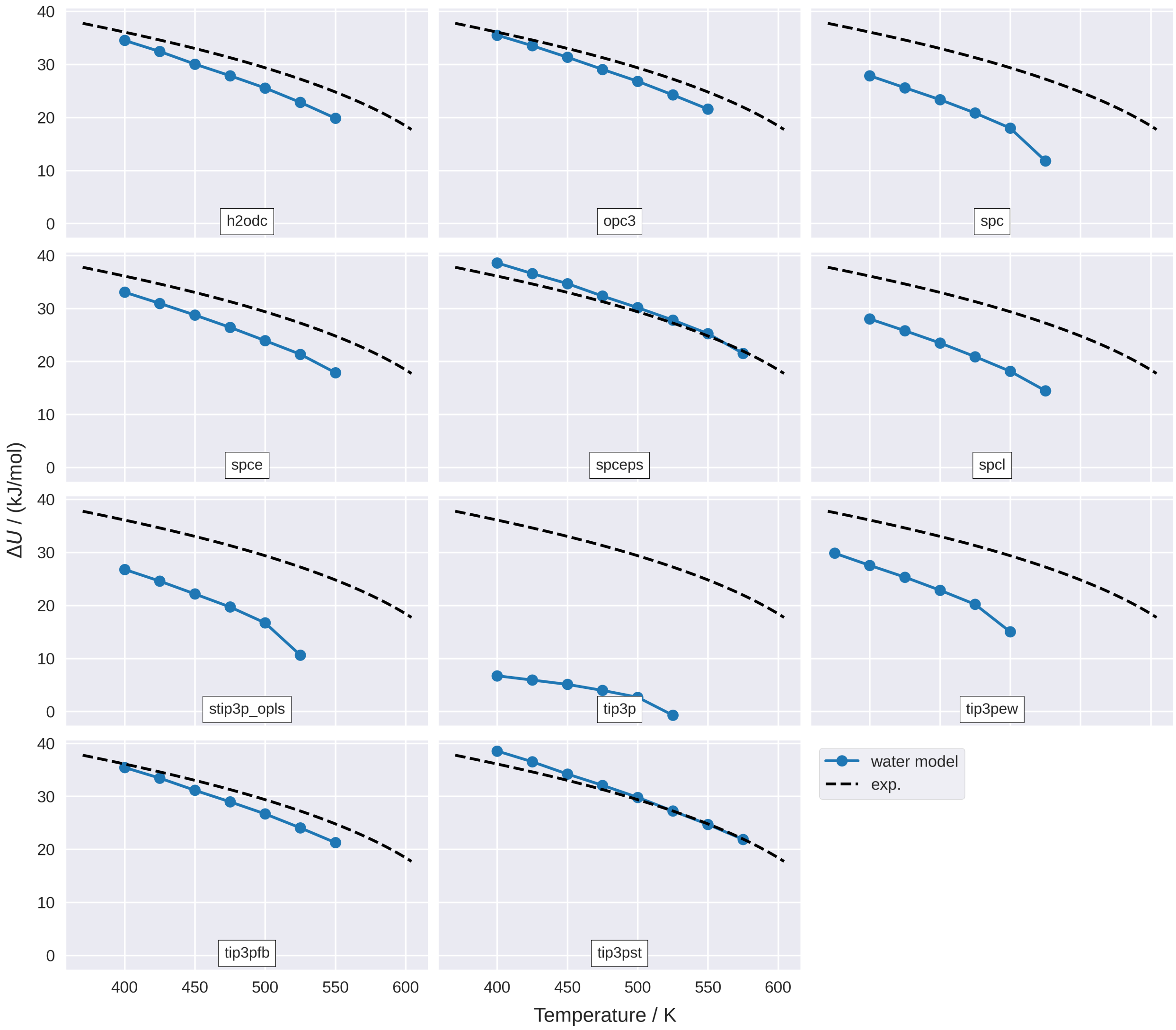

Figure S2: Difference in inner energy of vapor and liquid, ΔU, at different temperatures for different water models.

C.2 Volume Work

Below, we show our estimation of the volume work during evaporation at different temperatures for different water models. These values have been obtained from the densities in the corresponding coexistence simulation. Furthermore, we calculated the pressure from the corresponding canonical simulation of pure vapor.

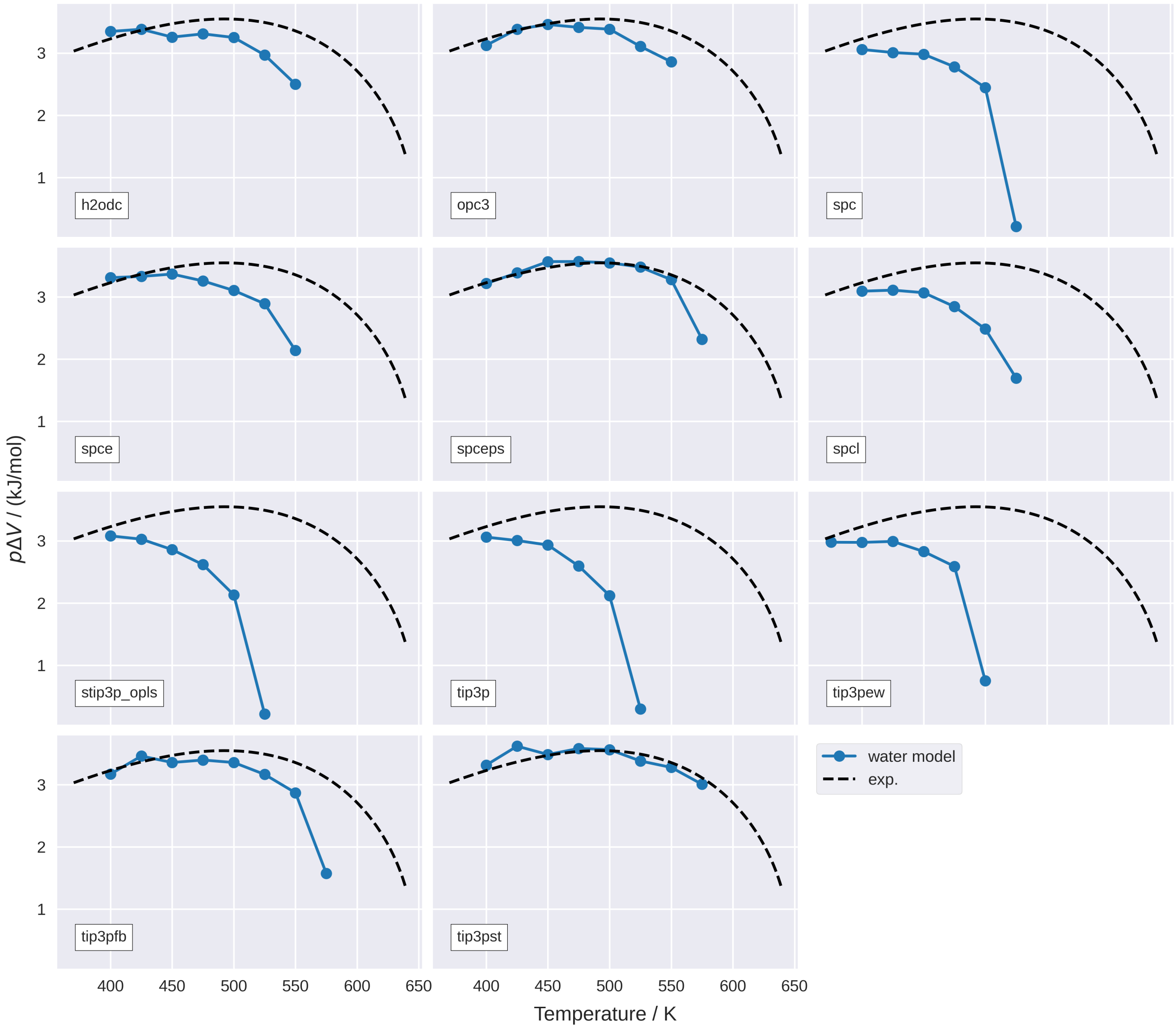

Figure S3: Volume work during evaporation, $p\Delta V$, at different temperatures for different water models.

D Temperature of Highest Density

Below, we show the density of liquid water obtained with various rigid 3-point water models at temperatures below 325 K. These values have been obtained from NPT simulations at p = 1 bar with 216 water molecules.

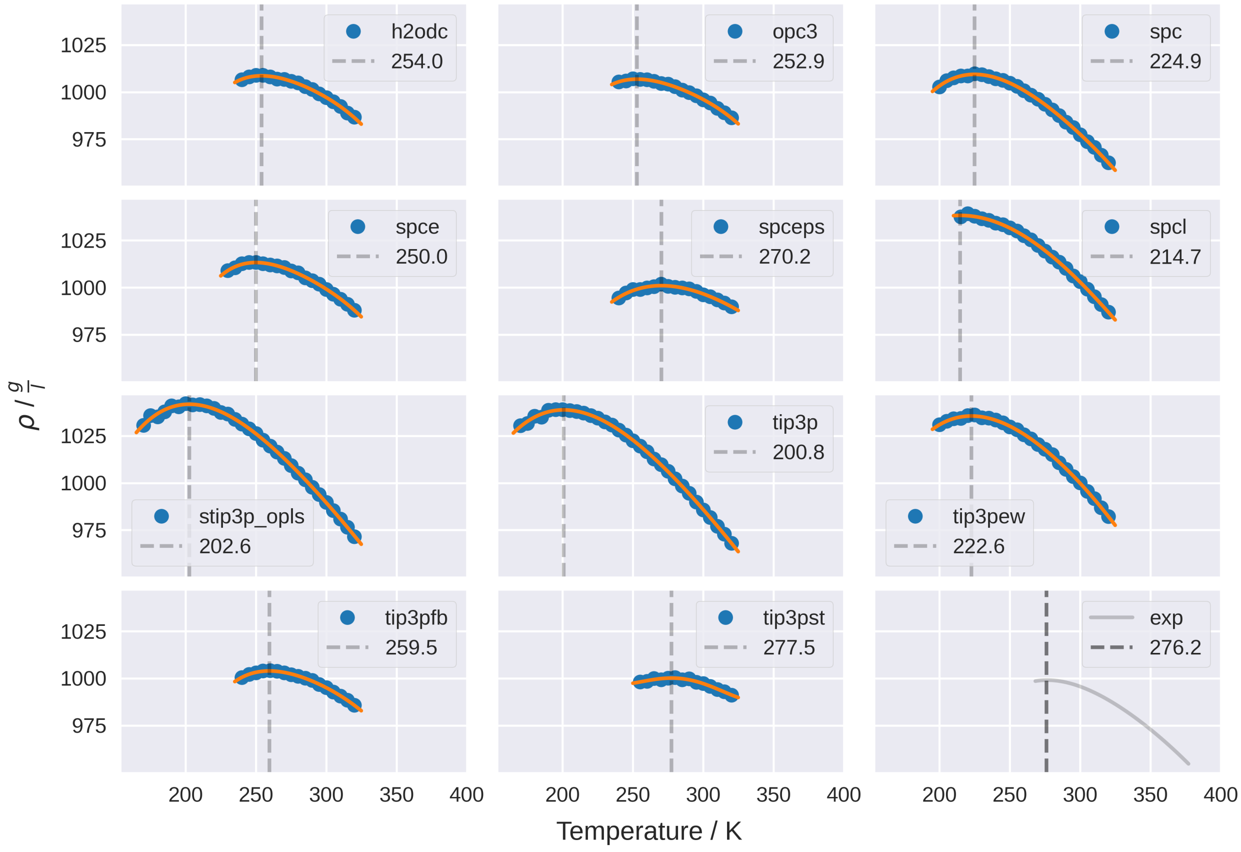

Figure S4: Densities of water, ρ, at low temperatures (i.e., below 325 K). Here, we estimated the temperature of highest densities, T_{MD}. These results refer to a pressure of p = 1 bar (also for the experimental measurements).

E Correlations

In this section, we show additional correlation plots that did not find space in the main manuscript.

E.1 T_{evap} vs. T_C

Below, we show the correlation between the temperature of spontaneous evaporation, T_{evap}, and the critical temperature, T_C. We found that these two characteristic temperatures correlate very well. According to our estimates, the relation between these two temperatures may be approximated by an offset of $\sim$ 26 K.

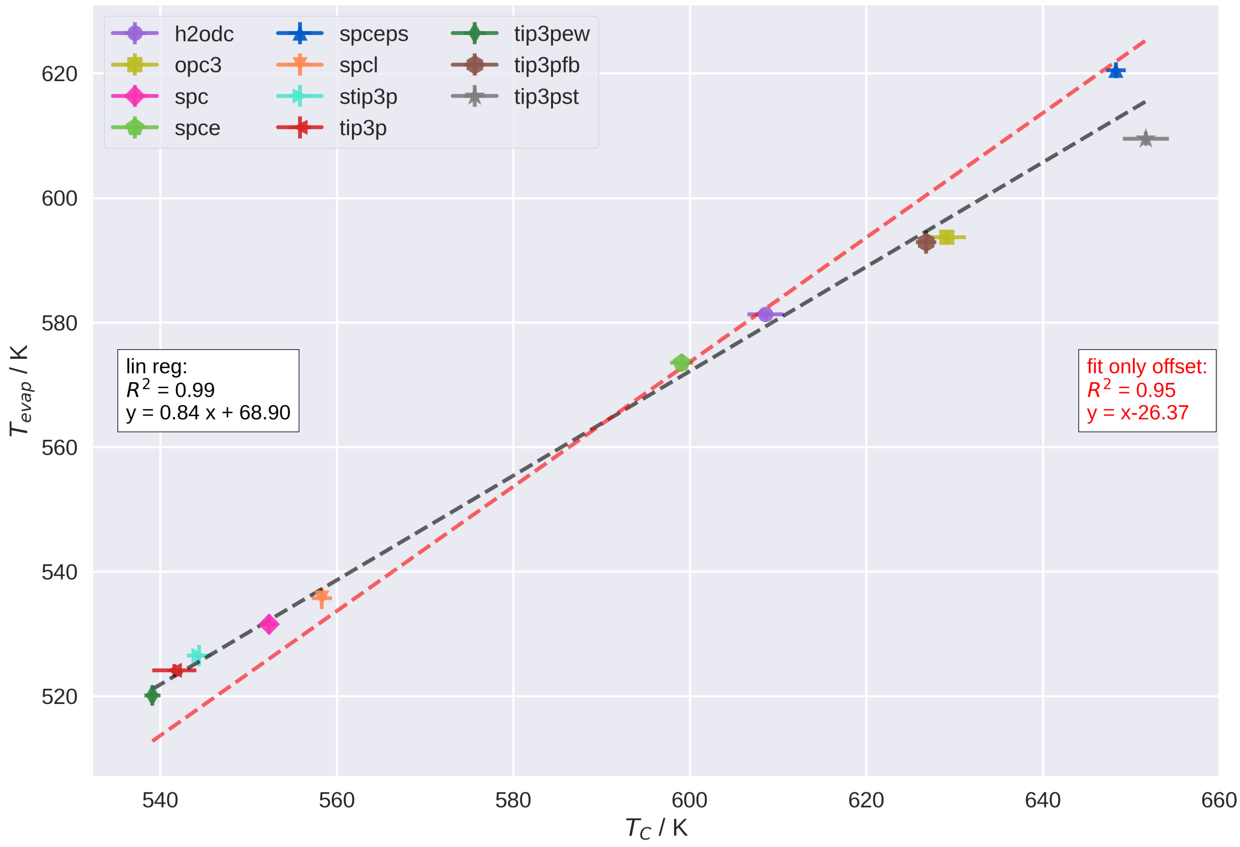

Figure S5: T_{evap} vs. T_C. Temperatures are given in Kelvin. We show different water models in different colors and different symbols. We performed two different kinds of linear fit (shown as dashed lines): Firstly, a full fit, i.e., with slope unequal to 1 (shown in gray). Secondly, fitting only the offset (shown in red).

E.2 T_C vs. T_{MD}

Below, in Figure S6, we show the correlation between the critical temperature, T_C, and the temperature of maximum density, T_{MD}. While we found a decent correlation when only fitting an offset between T_C and T_{MD}, performing a full linear fit yields significantly better correlation.

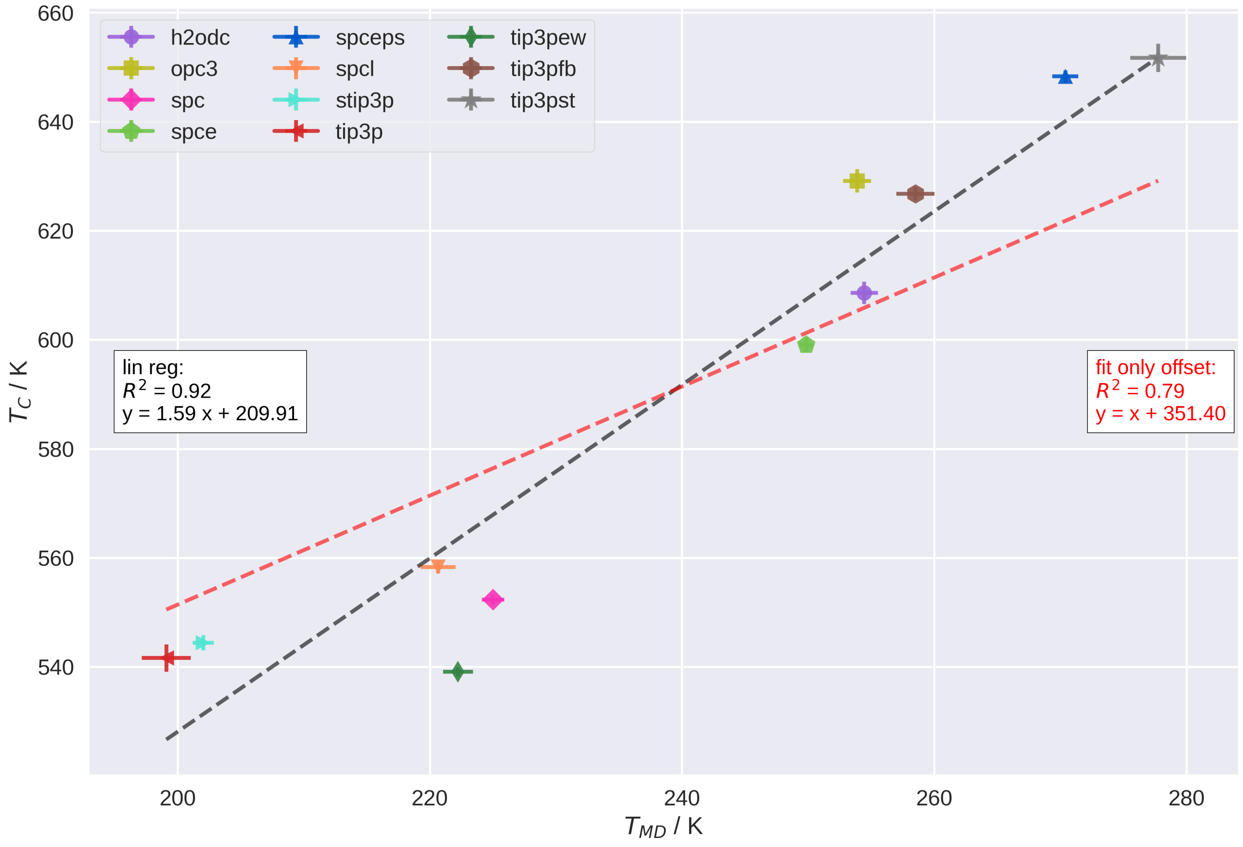

Figure S6: T_C vs T_{MD}. Temperatures are given in K. We show different water models in different colors and different symbols. We performed two different kinds of linear fit (shown as dashed lines): Firstly, a full fit, i.e., with slope unequal to 1 (shown in black). Secondly, fitting only the offset (shown in red).

E.3 T_{evap} vs. T_{MD}

Below, we show the correlation between the temperature of spontaneous evaporation, T_{evap}, and the temperature of maximum density, T_{MD}. While the correlation between these two temperatures is somewhat better, if a full linear fit is performed, only fitting an offset is apparently not much worse.

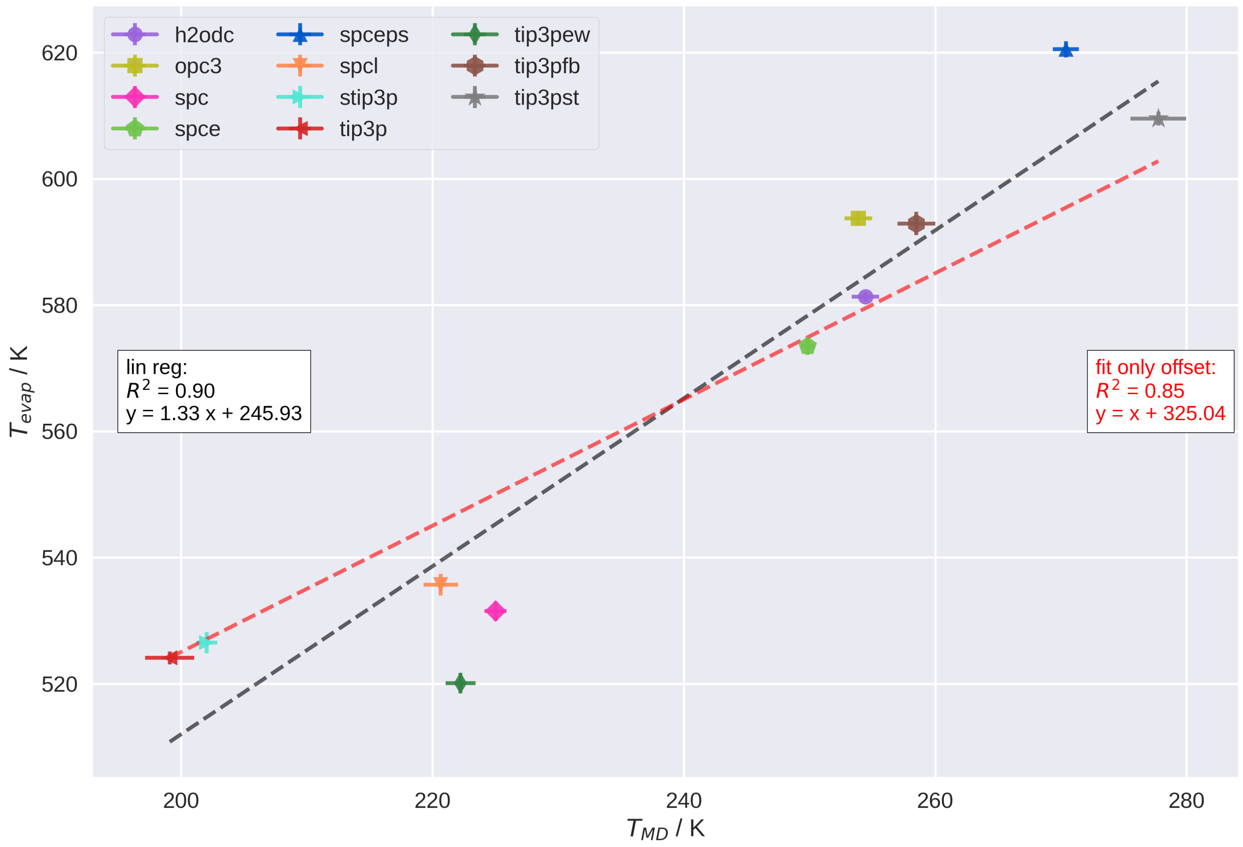

Figure S7: T_{evap} vs T_{MD}. Temperatures are given in K. We show different water models in different colors and different symbols. We performed two different kinds of linear fit (shown as dashed lines): Firstly, a full fit, i.e., with slope unequal to 1 (shown in black). Secondly, fitting only the offset (shown in red).

E.4 Correlation to Model Parameters

Below, we show the correlation between the temperature of spontaneous evaporation, T_{evap}, and model parameters of the here-used rigid 3-point water models. We found that these parameters generally do not show very high correlation. We only find weak trends for the LJ parameter σ_O and the angle ϕ_{HOH}.

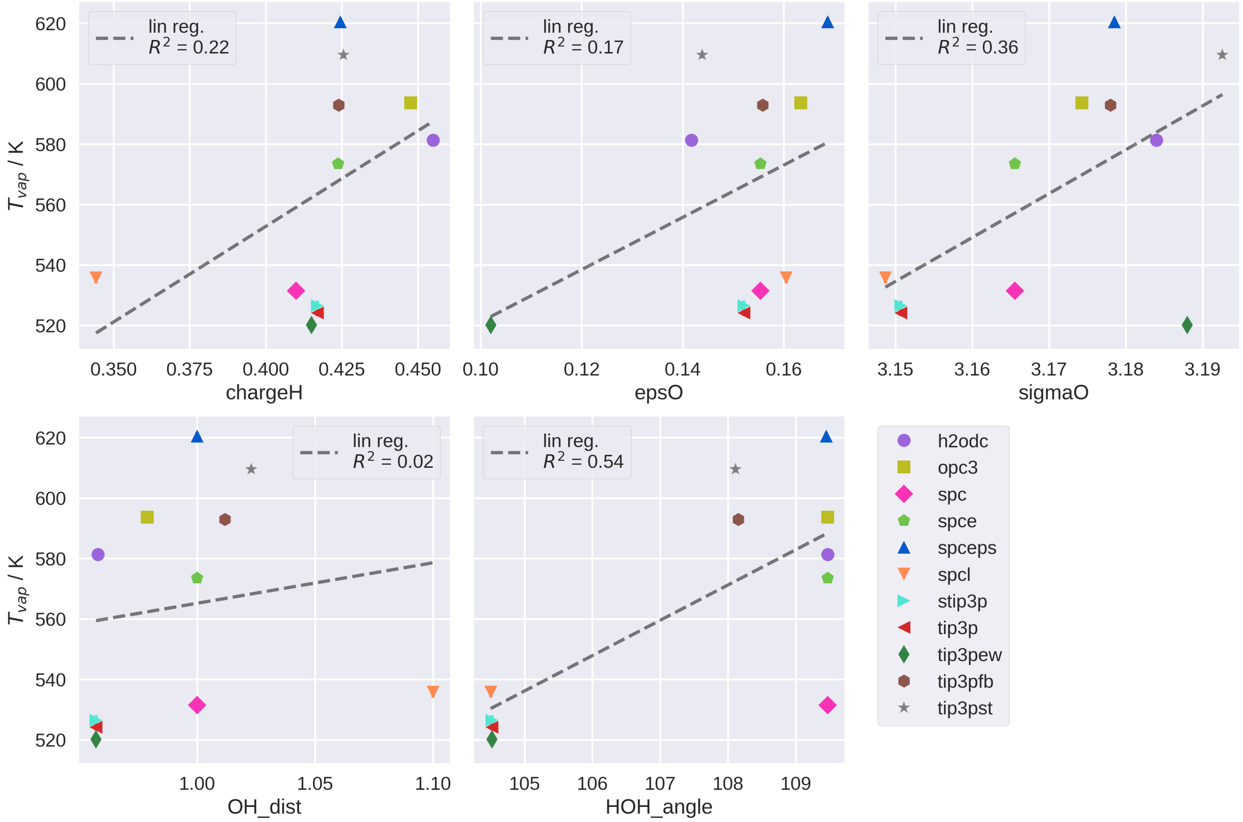

Figure S8: Correlation between the water model parameters and the temperature of spontaneous evaporation, T_{evap}.

F Convergence of Estimated Values With Simulation Time

We tested the dependence of our estimation of T_{evap} on the simulation time. To this end, we repeatedly estimated T_{evap} with decreasing heat rate (which corresponds to longer simulation times per temperature step). We visualized the results in Figure S9. We found that the mean of T_{evap} converges at a simulation time of 5 ns per temperature step. We only

tested this dependence for SPC/ϵ and furthermore assumed that the time dependence of this estimate is similar for all other here-used water models. Besides that, we estimated this convergence with the C-rescale barostat, and assumed the convergence of the estimates with the other two barostats to show similar time dependence. Separate evaluation of the convergence with all combinations of water model and barostat were simply infeasible. Given the consistency and the good correlation between all our estimates, we are confident with this choice of heating rate.

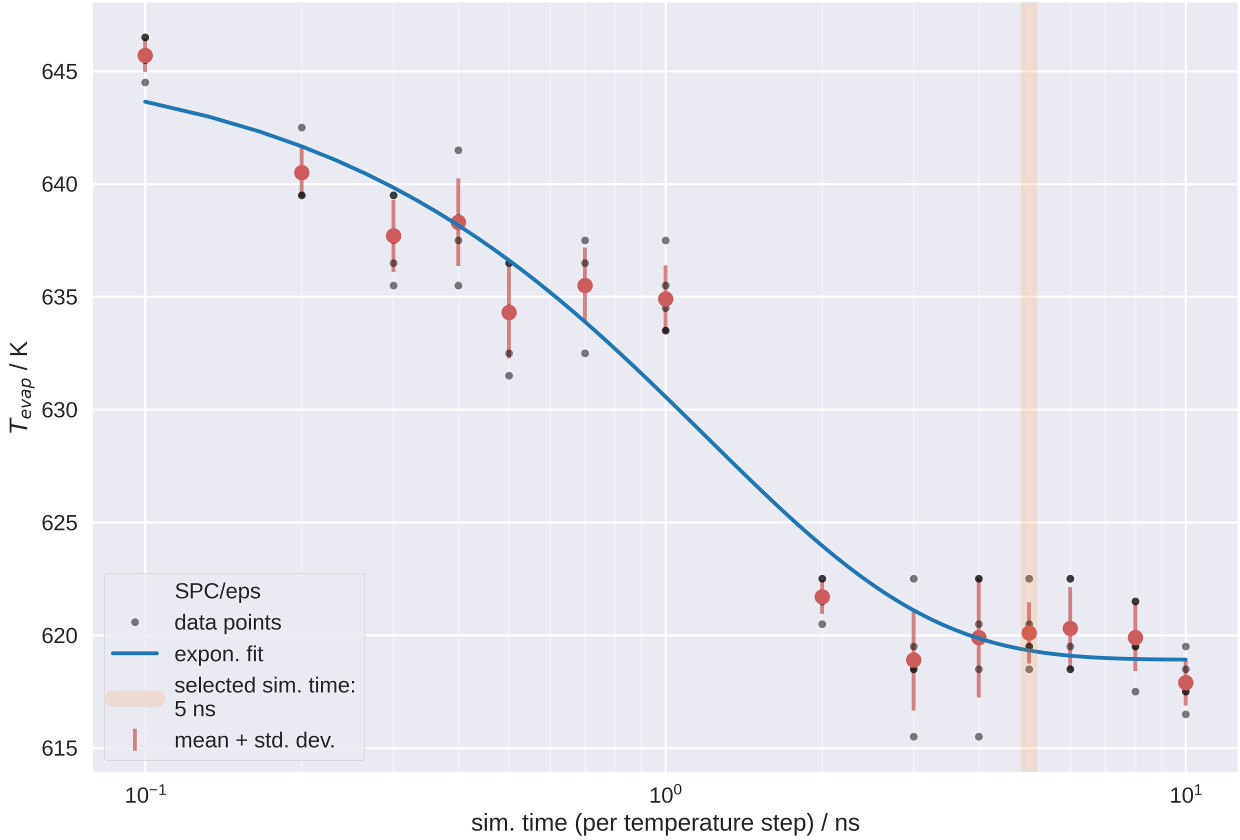

Figure S9: Dependence of the estimation of T_{evap} on the simulation time. We show mean and standard deviation of T_{evap} with increasing simulation time per temperature step. (This corresponds to decreasing heating rate). The here-shown data has been obtained with SPC/ϵ. According to this data, we chose a simulation time of 5 ns per temperature step for the studies of the other water models. Please note the logarithmic scale on the x-axis.
